# Performance of Diagnostic Guidelines in the Evaluation of Choledocholithiasis in Patients With Acute Biliary Presentation: A Systematic Review and Meta‐Analysis

**DOI:** 10.1002/wjs.12684

**Published:** 2025-06-26

**Authors:** Henry C. K. Kwok, Fransiska R. M. Falconer, Alain C. Vandal, Andrew G. Hill, Andrew D. Maccormick

**Affiliations:** ^1^ Department of Surgery Faculty of Medicine and Health Science The University of Auckland | Te Waipapa Taumata Rau Auckland New Zealand; ^2^ Counties Manukau District Health New Zealand Auckland New Zealand; ^3^ Department of Statistics Faculty of Science The University of Auckland | Te Waipapa Taumata Rau Auckland New Zealand

**Keywords:** choledocholithiasis, diagnosis, gallstone, guidelines, meta‐analysis, systematic review

## Abstract

**Background:**

Optimal management of acute biliary disease should include an assessment for possible choledocholithiasis (CBDS). Various diagnostic guidelines have been developed by expert bodies for this purpose, but uncertainties remain about their performance in wider practice.

**Methods:**

For this systematic review and meta‐analysis, we searched MEDLINE, Embase, and Scopus for studies on adult populations published in English language between 2000 and August 2024. All studies pertaining to the performance of diagnostic guidelines developed or adopted by regional, national, or international professional bodies are considered, but we excluded diagnostic tools or scoring systems developed locally with limited scopes, such as those employed by a single institution or a group of related institutions. We extracted or derived performance measures in the forms of true positive, true negative, false positive, and false negative and performed meta‐analysis using a multilevel random effects model to calculate pooled sensitivity and specificity for the reviewed guidelines and summarized their performance using summary ROC curves and AUCs. The quality of the evidence was assessed with the PROBAST risk of bias and applicability tool. This study is registered on PROSPERO (CRD42024581409).

**Findings:**

Of 1892 records identified, 31 studies were eligible with data available, all of which had a low to moderate risk of overall bias. All studies focused on one or more of three international guidelines, namely the ASGE guidelines in 2010, the revised ASGE guidelines in 2019, and the ESGE guidelines in 2019. For distinguishing patients at high risk for CBDS from those not at high risk, ASGE 2010, ASGE 2019, and ESGE guidelines have pooled sensitivities of 65% (CI: [57,73]), 63% (CI: [53,73]), and 62% (CI: [50,74]) and pooled specificities of 57% (CI: [48,66]), 75% (CI: [65,83]), and 82% (CI: [71,90]), respectively. For distinguishing patients at low risk for CBDS from those at greater than low risk, ASGE 2010, ASGE 2019, and ESGE guidelines have pooled sensitivities of 97% (CI: [92,99]), 95% (CI: [90,98]), and 84% (CI: [70,93]) and pooled specificities of 7% (CI: [3,18]), 11% (CI: [7,18]), and 15% (CI: [8,28]), respectively. Overall, the AUCs for ASGE 2010, ASGE 2019, and ESGE guidelines are 0.65, 0.74, and 0.73, respectively.

**Conclusion:**

ASGE 2019 and ESGE guidelines have comparable performance, with their key strength being the ability to rule out CBDS in low‐risk patients, allowing these patients to proceed with cholecystectomy without additional workup. All guidelines have limited specificity in identifying patients at high risk for CBDS and cannot reliably select patients for upfront ERCP.

**PROSPERO Registration:**

CRD42024581409.

## Graphical abstract

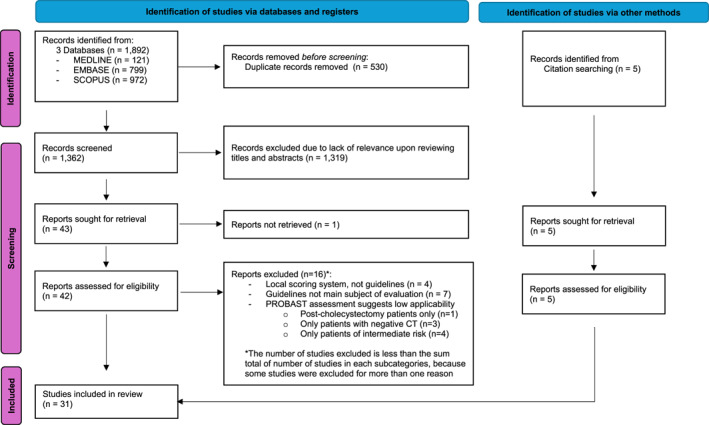

**Abbreviations**
ASGE: American Society of Gastrointestinal Endoscopy
AUC: area under curve
BSG: British Society of Gastroenterology
CBDS: choledocholithiasis
CI: 95% confidence interval
ERCP: endoscopic retrograde cholangiopancreatography
ESGE: European Society of Gastrointestinal Endoscopy
EUS: endoscopic ultrasound
FN: false negative
FP: false positive
IOC: intraoperative cholangiography
MRCP: magnetic resonance cholangiopancreatography
PROBAST: Prediction Model Risk of Bias Assessment Tool
ROC: receiver operating characteristic
SAGES: Society of American Gastrointestinal and Endoscopic Surgeons
WSES: World Society of Emergency Surgery
TN: true negative
TP: true positive

## 1 Background

Acute biliary disease is a common surgical problem. Choledocholithiasis, the presence of gallstone in common bile duct (CBDS), complicates 5%–20% of these presentations [1, 2, 3], potentially leading to increased morbidity and increased treatment complexity. CBDS can be identified with high accuracy with either endoscopic retrograde cholangiopancreatography (ERCP) prior to definitive treatment with cholecystectomy or during cholecystectomy with intraoperative cholangiography (IOC) [4, 5], but neither is routinely employed: In this case of ERCP, this is because it carries a risk of potentially serious adverse events such as pancreatitis and is usually performed on patients with high pretest probability [6, 7]. In the case of IOC, factors precluding its routine use include increased operation time, increased cost, and radiation exposure for both patients and staff [8, 9]. Magnetic resonance cholangiopancreatography (MRCP) and endoscopic ultrasound (EUS) are alternative modalities for diagnosing CBDS, with comparable diagnostic performance but without the issues associated with ERCP or IOC [10, 11]. Yet, these modalities are resource‐intensive, have limited availability, and may result in increased length of stay and additional financial costs [11].

Optimal management of acute biliary disease should incorporate an assessment for CBDS risk to select patients at elevated CBDS risk for these additional investigations (ERCP, IOC, MRCP, or EUS) while allowing patients at low risk to forego them safely. Over the years, various diagnostic guidelines have been proposed for this purpose, including those by the American Society of Gastrointestinal Endoscopy (ASGE) and the European Society of Gastrointestinal Endoscopy (ESGE) [5, 12, 13]. However, these guidelines are based on opinions from the expert panel drawing on low to moderate quality evidence only and are developed with their specific locality in mind. Uncertainties remain as to how these guidelines perform in wider, worldwide practice, and which of these, if any, represents the most performant strategy for stratifying CBDS risk.

The goal of this systematic review is to examine and compare the diagnostic performance of various published guidelines in the evaluation of CBDS.

## 2 Methods

### 2.1 Search Strategy

We have established our inclusion and exclusion criteria in accordance with the PRISMA 2020 checklist [14], with the review protocol registered at PROSPERO (CRD42024581409). On 22 August 2024, HK searched three healthcare‐related databases, the names, date coverages, and search strategies, which are as presented in Table 1. We imported and organized the search results in Rayyan [15], with which duplicated results were automatically removed. Two reviewers (HK and FF) independently screened titles and abstracts of all the studies retrieved to identify those which evaluated the guidelines in terms of diagnostic performance for a full text review. Discordances between HK and SF were resolved by consensus, or with adjudication by AM if a consensus could not be reached.

**TABLE 1:** Summary of literature search.

Database	Coverage	Search strategy	Reports retrieved
OVID Medline	2000‐current	exp choledocholithiasis/ choldocholithiasis.mp. common bile duct stone*.mp. CBD stone*.mp common bile duct calcul*.mp. CBD calcul*.mp 1 or 2 or 3 or 4 or 5 or 6 exp practice guideline/or guideline*.mp or exp guideline/ 1 and 8 Restricted to adult and English language Excluded conference abstracts	121
OVID Embase	2000‐current	choldocholithiasis.mp or exp common bile duct stone/ common bile duct stone.mp or exp common bile duct stone/ common bile duct calcul*.mp. [mp=title, abstract, heading word, drug trade name, original title, device manufacturer, drug manufacturer, device trade name, keyword heading word, floating subheading word, and candidate term word] 1 or 2 or 3 guideline*.mp. or exp practice guideline/6. 4 and 5 Restricted to adults and English language Excluded conference abstracts	799
Scopus	2000‐current	(ALL (“choledocholithiasis”) OR ALL (“CBD stone*”) OR ALL (“CBD calcul*”) OR ALL (“Common bile duct stone*”) OR ALL (“Common bile duct calcul*”)) AND TITLE‐ABS‐KEY (guideline*) Restricted to adults and English language Excluded conference abstracts	972

Next, HK performed a full text review of each screened study to (i) determine if the study was to be included or excluded and to (ii) extract data using a proforma if the study was included (see below). Decisions for inclusion or exclusion were independently verified by AM with disagreement resolved by consensus. HK also carried out a “snowball” search of the reference lists of the included studies to identify additional relevant studies; these additional studies were subjected to the same verification process by AM before the final inclusion.

### 2.2 Study Selection Criteria

In this review, we considered diagnostic guidelines identified by the search if they were developed or adopted by regional, national, or international (defined as at least state, provincial, or equivalent level) professional bodies or expert panels; specifically, we excluded diagnostic tools, algorithms, or scoring systems which were based on limited populations such as those devised and used locally by single institutions, due to uncertainties about their generalizability. Studies that used the diagnostic guidelines for patient selection but focused on outcomes other than performance of the guidelines were excluded. For example, we excluded studies primarily comparing the performance of MRCP or EUS on patients stratified as intermediate risk of CBDS using the guidelines.

For inclusion, the studies were required to report the diagnostic performance in terms of true positive (TP), true negative (TN), false positive (FP), and false negative (FN) to allow the construction of the 2 × 2 error matrix, or provide sufficient data for their derivation if these measures were not directly reported. Studies published as abstracts only were excluded. Non‐English language studies were excluded, unless official English versions were available.

### 2.3 Data Extraction

We designed an electronic proforma for data extraction from the eligible studies. Data extracted included

- Study country or region
- Study setting, for example, community hospital, tertiary center
- Study period
- Study design, for example, prospective versus retrospective
- Guidelines evaluated
- Gold standard(s) against which performance of guidelines was assessed
- Patient source
- Inclusion and exclusion criteria
- Total number of patients, number of patients with and without CBDS
- Diagnostic performance measures, either as reported or derived

### 2.4 Quality Assessment

Two investigators (HK and AM) evaluated the methodologies of the included studies for risk of bias and applicability using PROBAST, which covers 4 domains: selection of participants, predictors of assessment, outcomes of assessment, and analysis [16, 17]. Disagreements between HK and AM were resolved by consensus.

### 2.5 Data Analysis

For diagnostic guidelines which predict outcomes in a dichotomous fashion (i.e., CBD present or absent), we recorded their diagnostic performance as TP, TN, FP, and FN in the standard 2 × 2 error matrix. For diagnostic guidelines which stratify patients into more than 2 (or “*n*”) risk categories, they are treated as predictive tools with “*n*−1” diagnostic thresholds and their performances are recorded as “*n*−1” sets of TP, TN, FP, and FN using “*n*−1” thresholds. For example, because ESGE guidelines classify patients as high risk, intermediate risk, and low risk for CBDS, its diagnostic performance is recorded as two sets of TP, TN, FP, and FN, one for discriminating high risk from non‐high (i.e., intermediate and low) risk and one for discriminating low risk from greater than low (i.e., high and intermediate) risk.

We performed the meta‐analysis of the diagnostic performance of the guidelines using the “diagmeta” package [18] in the R environment version 4.4.1 [19]. This is based on a meta‐analysis approach by Steinhauser and colleagues [20]. Briefly, this approach is a meta‐regression model where the log‐odds of the proportion of TN over all negatives (specificity) and the log‐odds of the proportion of FN over all positives (1‐sensitivity) are modeled for each study at each threshold using a linear mixed effects model. The model uses distinct fixed effects (intercept and threshold slope parameters) for the negatives and positives, with the dependency between observations from the same study accounted for by random effects on the intercept and slope. Consequently, heterogeneity can be estimated for each threshold as the proportion of variance arising between studies at the threshold over the full variance, with higher values indicating greater heterogeneity. Four configurations for the random effects are possible, depending on whether they are distinct or similar between positives and negatives; we selected the configuration by minimizing the Bayesian information criterion in each set of the guidelines [21]. Fitted values from the model enable the recreation of a summary receiver operating curve (SROC) and the computation of the area under the curve (AUC), with approximate confidence intervals computed for these quantities using the delta method. Confidence intervals for the heterogeneity measures were obtained from 4000 bootstrap samples [22].

We evaluated outliership by comparing the absolute studentized residuals with the 97.5 centile of a normal distribution. We deemed a study an outlier if its absolute residual exceeded this centile at any threshold and removed it from the dataset for sensitivity analysis. Finally, we assessed the guidelines for equality of the parameters defining the summary ROC by concatenating the data from all guidelines and testing for an interaction of those parameters with the guidelines using a likelihood ratio test. We performed this test for all three guidelines simultaneously and for each pair of guidelines. The ROC curves from each guideline were estimated from the joint model involving all three guidelines and plotted together for comparison.

## 3 Results

### 3.1 Search Results

**FIGURE 1:**
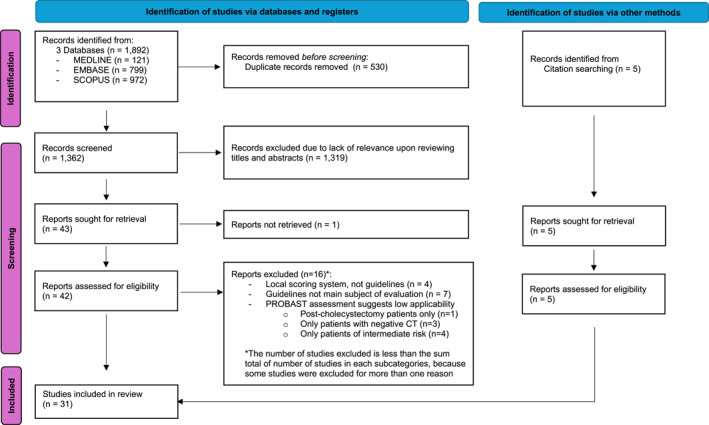
PRISMA flowchart.

The outcome of the literature search is summarized in a PRISMA flowchart (Figure 1). Briefly, we have retrieved 1892 studies from our initial search across 3 databases of which 530 were removed as duplicates semiautomatically using Rayyan. The titles and abstracts of the remaining 1362 articles were screened by HK and SF, resulting in the exclusion of 1319 articles for lack of relevance. Upon the full text review on the remainder 43 articles, we excluded 17 additional studies, including 4 studies examining local scoring systems or local adaptations of known guidelines with limited generalizability, 7 studies where guidelines were not main subjects of evaluation, 5 studies for low applicability upon PROBAST assessment, and 1 article for which the full text could not be retrieved. Therefore, the primary database search has resulted in 26 studies for inclusion. We performed “snowball” bibliography search of these studies concurrently with data extraction, identifying 5 additional articles for inclusion, three of which are non‐English articles with official English translations.

In all, 31 studies are included in this systematic review, the majority (*n* = 27) of which are retrospective. All have examined one or more of three international guidelines, namely the original ASGE guidelines in 2010, the revised ASGE guidelines in 2019, and the ESGE guidelines in 2019. There is one study reporting on the British Society of Gastroenterology (BSG) guidelines, one on the Society of American Gastrointestinal and Endoscopic Surgeons (SAGES) guidelines, and one on the World Society of Emergency Surgery (WSES) guidelines. For completeness, we have included qualitative descriptions of these guidelines but they are not evaluated with meta‐analytic technique due to limited data available.

A summary of the included studies, including their characteristics and an assessment of their risk of bias and applicability as per PROBAST, is presented in Tables 2 and 3.

**TABLE 2:** Summary of the included studies.

First author	Country	Study period	Patients evaluated	Guidelines evaluated	No of patients	%CBDS
Adams et al. [23]	USA	2007–2012	All suspected of CBDS	ASGE 2010	498	42%
Blum et al. [24]	Australia	2018–2023	All suspected of CBDS	ASGE 2019	222	51%
Gastelbondo‐Morales et al. [25]	Columbia	2017–2018	All suspected of CBDS	ASGE 2010, BSG	145	86%
Hasak et al. [26]	USA	2013–2019	All suspected of CBDS	ASGE 2010 & 2019	1098	66%
He et al. [27]	China	2011–2013	All suspected of CBDS	ASGE 2010	2724	40%
Jacob et al. [28]	USA	2012–2017	All suspected of CBDS	ASGE 2010 & 2019	267	72%
Ovalle‐Chao et al. [29]	Mexico	2016–2019	All suspected of CBDS	ASGE 2019	347	72%
Silva‐Santisteban et al. [30]	USA	2021–2022	All suspected of CBDS	ASGE 2019	359	63%
Steinway et al. [31]	USA	2009–2016	All suspected of CBDS	ASGE 2010 & 2019, ESGE	1378	59%
Suarez et al. [32]	USA	2009–2014	All suspected of CBDS	ASGE 2010	173	41%
Tunruttanakul et al. [33]	Thailand	2019–2021	All suspected of CBDS	ASGE 2019, ESGE	521	61%
Wangchuk and Srichan [34]	Thailand	2019–2020	All suspected of CBDS	ASGE 2019, ESGE, SAGES	280	76%
Zhang et al. [35]	China	2011–2018	All suspected of CBDS	ASGE 2010 & 2019, ESGE	1199	57%
Černe et al. [36]	Solvenia	2020–2020	Pancreatitis	ASGE 2019, ESGE	86	34%
Gouveia et al. [37]	Portugal	2012–2016	Cholecystitis	ASGE 2010	40	45%
Jagtap, HS et al. [38]	India	2016–2018	Pending cholecystectomy	ASGE 2019, ESGE	1042	26%
Lai et al. [39]	Taiwan	2017–2017	Suspected CBDS & IOC	WSES	990	20%
Reddy [40]	India	2018–2019	Cholecystitis	ASGE 2019, ESGE	173	36%
Tintara et al. [41]	USA	2008–2018	Pancreatitis	ASGE 2010 & 2019	156	58%
Toro‐Calle et al. [42]	Columbia	2017–2017	Pending cholecystectomy	ASGE 2010	424	22%
Woo et al. [43]	Korea	2009–2021	Had cholecystectomy	ASGE 2019	1223	23%
Nárvaez‐Rivera [44]	Mexico	2012–2014	High/intermediate risk	ASGE 2010	256	57%
Sadeghi et al. [45]	Iran	2020–2021	High/intermediate risk	ASGE 2019	124	56%
Chandran et al. [46]	USA	2013–2019	Suspected CBDS & ERCP	ASGE 2010 & 2019	744	73%
Dalai et al. [47]	USA	2015–2019	Suspected CBDS & ERCP	ASGE 2019	52	85%
Ebrahim et al. [48]	Denmark	2011–2012	Suspected CBDS & ERCP	ASGE 2010	186	75%
Kuzu et al. [49]	Turkey	2010–2014	Suspected CBDS & ERCP	ASGE 2010	888	79%
Magalhães [50]	Portugal	2010–2013	Suspected CBDS & ERCP	ASGE 2010	268	67%
Rubin et al. [51]	USA	2007–2010	Suspected CBDS & ERCP	ASGE 2010	521	56%
Sethi et al. [52]	USA	2011–2012	Suspected CBDS & ERCP	ASGE 2010	336	68%
Singhvi et al. [53]	USA	2009–2009	Suspected CBDS & ERCP	ASGE 2010	38	47%

**TABLE 3:** Assessment of risk of bias and applicability by PROBAST.

	Risk of bias				Applicability			Overall	
First author	Participants	Predictors	Outcome	Analysis	Participants	Predictors	Outcome	Risk of bias	Applicability
Adams et al. [23]	+	+	+	C	+	+	+	+	+
Blum et al. [24]	+	+	+	C	+	+	+	+	+
Gastelbondo‐Morales et al. [25]	+	+	+	C	+	+	+	+	+
Hasak et al. [26]	+	+	+	C	+	+	+	+	+
He et al. [27]	+	+	+	C	+	+	+	+	+
Jacob et al. [28]	+	+	+	C	+	+	+	+	+
Ovalle‐Chao et al. [29]	+	+	+	C	+	+	+	+	+
Silva‐Santisteban et al. [30]	+	+	+	C	+	+	+	+	+
Steinway et al. [31]	+	+	+	C	+	+	+	+	+
Suarez et al. [32]	+	+	+	C, D	+	+	+	+	+
Tunruttanakul et al. [33]	+	+	+	C	+	+	+	+	+
Wangchuk and Srichan [34]	+	+	+	C	+	+	+	+	+
Zhang et al. [35]	+	+	+	C	+	+	+	+	+
Černe et al. [36]	A	+	+	C, D	A	+	+	A	A
Gouveia et al. [37]	A	+	+	C, D	A	+	+	A	A
Jagtap, HS et al. [38]	A	+	+	C	A	+	+	A	A
Lai et al. [39]	B	+	+	C	B	+	+	B	B
Reddy [40]	A	+	+	C, D	A	+	+	A	A
Tintara et al. [41]	A	+	+	C, D	A	+	+	A	A
Toro‐Calle et al. [42]	A	+	+	C, D	A	+	+	A	A
Woo et al. [43]	B	+	+	C	B	+	+	B	B
Nárvaez‐Rivera [44]	+	+	+	C, E	+	+	+	+	E
Sadeghi et al. [45]	+	+	+	C, E	+	+	+	+	E
Chandran et al. [46]	B	+	+	C	B	+	+	B	B
Dalai et al. [47]	B	+	+	C, D	B	+	+	B	B
Ebrahim et al. [48]	B	+	+	C	B	+	+	B	B
Kuzu et al. [49]	B	+	+	C	B	+	+	B	B
Magalhães [50]	B	+	+	C	B	+	+	B	B
Rubin et al. [51]	B	+	+	C	B	+	+	B	B
Sethi et al. [52]	B	+	+	C	B	+	+	B	B
Singhvi et al. [53]	B	+	+	C, D	B	+	+	B	B

*Note:* +: Low risk of bias/low concern for applicability; −: high risk of bias/high concern for applicability; A: probably low risk of bias/probably low concern for applicability because studies included subgroups of patients with certain clinical characteristics only, for example, pancreatitis, cholecystitis; B: probably low risk of bias/probably low concern for applicability because studies included subgroups of patients who had EUS/ERCP, IOC, or cholecystectomy only; C: probably low risk of bias/probably low concern for applicability because patients were excluded when there was insufficient data to apply the guidelines retrospectively. This was either stated in the study methodology or implied; D: probably low risk of bias/probably low concern for applicability because studies involved less than 100 participants; E: probably low risk of bias/probably low concern for applicability because studies excluded patients classified as low risk by the guidelines and only evaluated those at intermediate or high risk.

### 3.2 Performance of ASGE 2010, ASGE 2019, and ESGE Guidelines

In ASGE 2010, ASGE 2019, and ESGE guidelines, patients are stratified into high risk, intermediate risk, or low risk for CBDS based on various clinical, biochemical, and ultrasound criteria, as presented in Table 4. All three guidelines recommend ERCP or laparoscopic treatment for CBDS for patients stratified as high risk, MRCP or EUS for patients stratified as intermediate risk, and no additional workup prior to cholecystectomy for patients stratified as low risk. Given these similarities, we have applied identical strategies when performing the meta‐analysis of these guidelines by treating these three‐tiered stratification systems as predictive tools with two distinct thresholds, as detailed in the “Data Analysis” section above.

**TABLE 4:** Summary of ASGE 2010, ASGE 2019 and ESGE guidelines.

Guidelines	Low‐risk criteria	Intermediate risk criteria	High risk criteria	Recommendations
ASGE 2010 [5]	No high or intermediate risk factors	Age > 55^a^ Or clinical pancreatitis Or abnormal liver function tests Or bilirubin 1.8–4 mg/dL^b^ OR CBD dilatation on ultrasound (but not both)	Clinical cholangitis Or CBDS on ultrasound Or bilirubin > 4 mg/dL^a^ Or bilirubin 1.8–4 mg/dL^b^ AND CBD dilatation on ultrasound	Laparoscopic cholecystectomy for low risk EUS or MRCP for intermediate risk ERCP for high risk
ASGE 2019 [12]	No high or intermediate risk factors	Age > 55^a^ Or abnormal liver function tests Or CBD dilatation on ultrasound	Clinical cholangitis Or CBDS on imaging Or bilirubin > 4 mg/dL^a^ AND CBD dilatation on ultrasound	
ESGE [13]	Normal liver function tests And normal CBD on ultrasound	Abnormal liver function tests or CBD dilatation on ultrasound	Clinical cholangitis Or CBDS on ultrasound	

^a^ Criteria included in ASGE 2010 and ASGE 2019 guidelines not included in ESGE guidelines.

^b^ Criteria included in ASGE 2010 guidelines and not in ASGE 2019 or ESGE guidelines.

**FIGURE 2:**
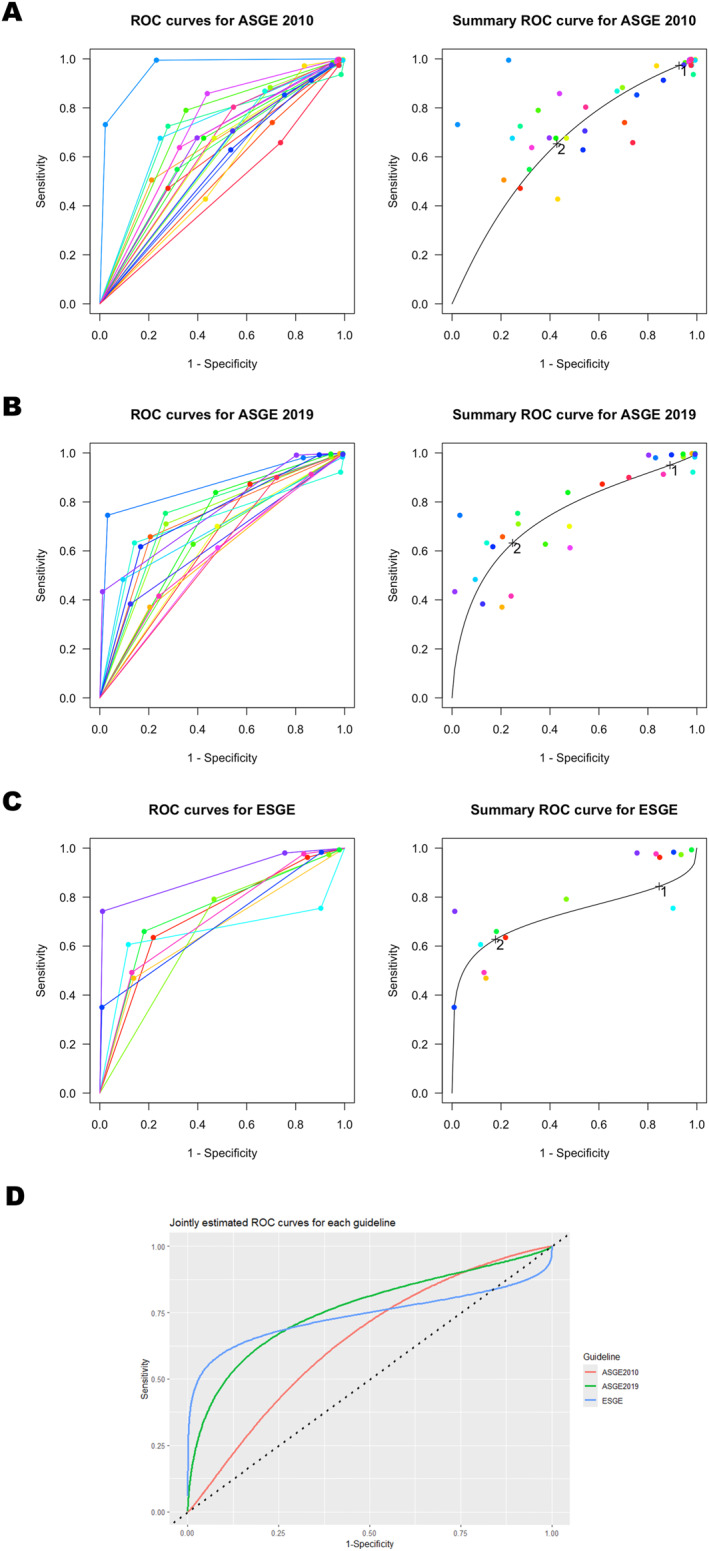
ROC and summary ROC curves for (A) ASGE 2010 guidelines, (B) ASGE 2019 guidelines, and (C) ESGE guidelines. Label “1” denotes pooled sensitivity/specificity of the guidelines when they are used to differentiate patients at low risk from patients at greater than low risk for CBDS. Label “2” denotes pooled sensitivity/specificity of the guidelines when they are used to differentiate patients at high risk from patients at less than high risk for CBDS. (D) Graph of the summary ROC curves for the three guidelines using a jointly estimated model.

Data for ASGE 2010, ASGE 2019, and ESGE guidelines are available from 21 studies with 12,047 patients, 17 studies with 9271 patients, and 8 studies with 5028 studies, respectively. For distinguishing patients at high risk for CBDS from those not at high risk, ASGE 2010, ASGE 2019, and ESGE guidelines have pooled sensitivities of 65% (CI: [57,73]), 63% (CI: [53,73]), and 62% (CI: [50,74]) and pooled specificities of 57% (CI: [48,66]), 75% (CI: [65,83]), and 82% (CI: [71,90]), respectively. For distinguishing patients at low risk for CBDS from those greater than low risk, ASGE 2010, ASGE 2019, and ESGE guidelines have pooled sensitivities of 97% (CI: [92,99]), 95% (CI: [90,98]), and 84% (CI: [70,93]) and pooled specificities of 7% (CI: [3,18]), 11% (CI: [7,18]), and 15% (CI: [8,28]), respectively. Overall, the AUCs for ASGE 2010, ASGE 2019, and ESGE guidelines are 0.65, 0.74, and 0.73 respectively, with their ROCs and summary ROCs as presented in Figure 2A–C. Test of similarity suggests the guidelines are dissimilar (*p* = 0.000001 for the overall comparison between three guidelines and *p* < 0.02 for all pairwise comparisons between guidelines). The jointly estimated ROC curves are presented in Figure 2D.

Results of heterogeneity analysis for each guideline at each of the diagnostic threshold are presented in Table 5. Of note, studies evaluating the ASGE 2010 guideline for its ability to discriminate patients with low risk from those with non‐low risk for CBDS have appreciably higher between‐study heterogeneity (0.83 of total variance), when compared to other studies examining the other diagnostic threshold or studies examining the ASGE 2019 and ESGE guidelines (all < 50% of total variance). Upon outliership assessments, we have identified two studies evaluating ASGE 2010 guidelines [27, 42], two studies evaluating ASGE 2019 guidelines [38, 43], and two studies evaluating ESGE 2019 guidelines [35, 38], where their performance measures depart appreciably from the remaining included studies. Notwithstanding, there are only small absolute changes to the overall performance of the guidelines upon subgroup analysis after excluding these outliers, with AUCs of 0.71, 0.74, and 0.76 for ASGE 2010, ASGE 2019, and ESGE guidelines, respectively. The heterogeneity for ASGE 2010 guidelines at the low versus. non‐low risk threshold decreases to 0.59 (of total variance) upon exclusion of the outliers.

**TABLE 5:** Proportion of total mixed model variance explained by study heterogeneity.

	For discriminating between low versus. nonlow risk		For discriminating between nonhigh versus. high risk	
Guidelines	Proportion estimates	CI	Proportion estimates	CI
ASGE 2010	0.83	[0.50, 0.94]	0.25	[0.09, 0.62]
ASGE 2019	0.27	[0.10, 0.71]	0.45	[0.25, 0.75]
ESGE	0.34	[0.01, 0.81]	0.22	[0.02, 0.72]

### 3.3 Performance of Other Guidelines

Gastelbondos and colleagues reported the performance of BSG guidelines in 145 patients, with sensitivity of 65% and specificity of 33% for prediction of CBDS. Wangchuk and colleagues reported the performance of SAGES guidelines in 280 patients, with sensitivity of 81% and specificity of 72% for distinguishing those at high risk from non‐high risk and 98% and 12%, respectively, for distinguishing those at low risk from greater than low risk. The corresponding figures for WSES guidelines are 61%, 98%, 100%, and 40%, respectively, as reported by Lai and colleagues in 990 patients.

## 4 Discussion

In this systematic review, we have identified 6 diagnostic guidelines predicting CBDS in patients with acute biliary presentation. Of these, there is sufficient data in the literature for the meta‐analysis of the original ASGE guidelines in 2010, the revised ASGE guidelines in 2019, and the ESGE guidelines. All three guidelines stratify patients into high, intermediate, or low risk categories for CBDS, and are similar in their management recommendations.

When the guidelines are used to distinguish patients at low risk for CBDS from those at greater than low risk, both iterations of ASGE guidelines have excellent pooled sensitivities of 95%–97% for CBDS, while that of the ESGE guidelines is lower at 84%. The observed difference may reflect the inclusion of patients older than 55 as intermediate risk in both ASGE but not the ESGE guidelines, even though age was found to be a nonspecific and minimally predictive criterion in a meta‐analysis of predictor performance by Wang et al. [54]. These high sensitivities can be utilized to advantage clinically, because a low‐risk classification can reliably rule out most CBDS and allow low‐risk individuals to proceed to cholecystectomy without additional investigations. At this diagnostic threshold, all guidelines have poor specificities for CBDS from 7% to 15% only, resulting in many patients classified as at least intermediate risk thus prompting for additional investigations.

When the guidelines are used to identify individuals at high risk for CBDS for upfront ERCP, the ASGE 2019 and ESGE guidelines are more specific with pooled specificities of 75% and 82%, respectively, compared to a specificity of 57% only with the ASGE 2010 guidelines. The lower specificities of ASGE guidelines probably reflect the inclusion of elevated bilirubin as a high‐risk criterion, when bilirubin in isolation had been found to be an unreliable predictor for CBDS on meta‐analysis [54]. This is particularly relevant with ASGE 2010 guidelines, where a lesser degree of bilirubin elevation is considered high risk if the bile duct is also dilated. In use, the higher specificities of ASGE 2019 and ESGE guidelines translate to lower false positive rates of CBDS and therefore lower rates of nontherapeutic ERCP; however, this still amounts to 18% even with the most selective ESGE criteria, which comprise only of CBDS on ultrasound and acute cholangitis. In their meta‐analysis, Wang et al. found that CBDS on ultrasound is highly predictive for CBDS with an adjusted odds ratio of 8.6 and an average specificity of 94%, but acute cholangitis is much less predictive with an adjusted odds ratio of 2.3 and an average specificity of 89% [54]. We hypothesize that the modest specificity of the guidelines is probably partly due to the inclusion of acute cholangitis as a criterion, which is variably and perhaps less objectively diagnosed on clinical grounds [55]. In terms of sensitivities, all guidelines perform comparably but only modestly with pooled sensitivities of 62%–65%, resulting in over 30% of patients with CBDS misclassified as nonhigh risk. This is however of less clinical import because these individuals are likely to undergo further evaluation with MRCP or EUS with similar eventual outcomes, albeit at the price of additional costs and possible delays to definitive treatment.

Overall, ASGE 2019 and ESGE guidelines have similar performances and limitations with only minor differences in how they balance between sensitivity and specificity. Both perform better than ASGE 2010 guidelines, but neither is clearly superior with similar AUCs. In clinical practice, we believe the main value of the guidelines lies in their ability to rule out CBDS. For example, patients without any ASGE 2019 criteria (i.e., abnormal liver function, CBD > 6 mm, age > 55) have very low rate of CBDS and can proceed to cholecystectomy without additional workup. On the other hand, the guidelines have only modest specificity when used to identify patients at high risk for CBDS. Although the guidelines can help limit ERCP to a smaller group of patients if used as intended, the nontherapeutic ERCP rate is still arguably too high resulting in an undesirable number of nontherapeutic ERCP with its attending risks of ERCP‐related complications such as pancreatitis or perforation. A more refined strategy is therefore indicated.

With all guidelines, a substantial proportion of patients are expected to fall into the intermediate risk category, but there remains a debate on how these patients are best managed. Although much research effort has previously focused on comparing MRCP and EUS in this patient group [11, 56, 57, 58, 59], both ASGE and ESGE expert panels have deemed the evidence to be of low to moderate quality only [12, 13], citing a lack of data on cost‐effectiveness and other logistical considerations. To optimize the management of these patients, further research in these deficient areas is required. Moreover, alternative imaging techniques such as abbreviated MRCP [60] or novel tools such as machine learning [31, 47] have potentials to become disruptors to or even possible replacement for existing treatment paradigms, with additional avenues for improvement. For instance, Steinway et al. have demonstrated superior AUC with machine learning when compared to ASGE 2019 and ESGE guidelines in a proof‐of‐concept study [31].

This systematic review has two main limitations. First, the included studies have drawn on different patient sources which can be broadly divided into three categories: (i) patients with acute biliary presentation identified via multiple records, (ii) patients who have undergone ERCP or EUS identified from endoscopic database, and (iii) patients with certain clinical diagnosis such as pancreatitis. It is conceivable that patients from different sources may have different underlying characteristics, with perhaps the most important example being possible higher pretest probabilities for CBDS when patients were drawn from the ERCP database. However, we argue that this is unlikely to affect the outcome of the meta‐analysis because sensitivity and specificity, the two measures we have focused on, are intrinsic to the guidelines and are prevalence‐invariant. Moreover, although there is between‐study heterogeneity particularly with respect to the ASGE 2010 guidelines, our sensitivity analysis suggests the impact is small in magnitude; for instance, in the worst case of ASGE 2010, its AUC has changed from 0.65 to 0.71 only after excluding outliers. Second, we have opted to test the guideline similarity using the likelihood ratio test because included studies are shared between the guidelines making the data dependent. Although this test has indicated that the guidelines are dissimilar, we would like to note that (i) likelihood testing is inherently anticonservative and may overstate the dissimilarities and (ii) the absolute differences in AUCs between the guidelines, even if they exist, are small and likely of little clinical import. For the above reasons, we assert that these limitations are unlikely to materially impact our key observations or the overall conclusions we draw from them.

In summary, the ASGE 2019 and ESGE guidelines have comparable performance, with their key strength being the ability to rule out CBDS in low‐risk patients allowing these patients to proceed with cholecystectomy without additional workup. Both guidelines have only limited ability to identify patients at high risk for CBDS; therefore, using the guidelines to select patients for upfront ERCP is probably inappropriate. Both guidelines classify sizable proportions of patients as intermediate risk for whom management consensus is lacking and further research is required.

## Author Contributions

**Henry C. K. Kwok:** conceptualization, methodology, investigation, validation, visualization, project administration, formal analysis, writing – original draft, writing – review and editing, data curation, software. **Fransiska R. M. Falconer:** data curation, investigation, validation. **Alain C. Vandal:** investigation, validation, formal analysis, visualization, writing – review and editing, software, methodology. **Andrew G. Hill:** conceptualization, methodology, investigation, supervision, resources, writing – review and editing. **Andrew D. Maccormick:** conceptualization, methodology, data curation, investigation, validation, supervision, resources, writing – review and editing.

## Ethics Statement

The authors declare compliance with the ethical requirements of the Journal for the present manuscript.

## Conflicts of Interest

The authors declare no conflicts of interest.

## Data Availability

The data that support the findings of this study are available from the corresponding author upon reasonable request.
